# The interference with transapical transcatheter aortic valve implantation by the anterior chordae tendineae preserved at previous mitral valve replacement

**DOI:** 10.1002/ccr3.2406

**Published:** 2019-08-30

**Authors:** Akihisa Furuta, Katsumasa Sato, Hironobu Morimoto, Shogo Mukai, Kenji Goto

**Affiliations:** ^1^ Department of Cardiovascular Surgery Fukuyama Cardiovascular Hospital Hiroshima Japan; ^2^ Department of Cardiology Fukuyama Cardiovascular Hospital Hiroshima Japan

**Keywords:** mitral valve surgery, transapical transcatheter aortic valve implantation

## Abstract

Preserved anterior chordae tendineae is a possible risk factor for disturbing delivery of a transcatheter heart valve. Inserting a sheath just below the aortic valve for delivery of the transcatheter heart valve might be proposed as an alternative to avoid the chordae tendineae.

## 1 INTRODUCTION

A 91‐year‐old woman with a history of mitral valve replacement with a mechanical valve was diagnosed with severe aortic stenosis. She underwent transapical transcatheter aortic valve implantation, during which the device became trapped in left ventricle. The device was forced into the aortic valve and finally released.

Transcatheter aortic valve implantation (TAVI) has emerged as a valuable option for treating high‐risk patients with symptomatic aortic valve stenosis.^1^, ^2^, ^3^ Previous TAVI trials excluded patients with mitral prostheses because of concerns regarding interference with the transcatheter heart valve (THV). Although TAVI has recently been reported as safe and effective even in this complex setting, complications remain an issue in some cases.^4^, ^5^ Herein, we report a case of transapical TAVI after mitral valve replacement, during which the THV became stuck in the left ventricle.

## 2 CASE REPORT

**Figure 1:**
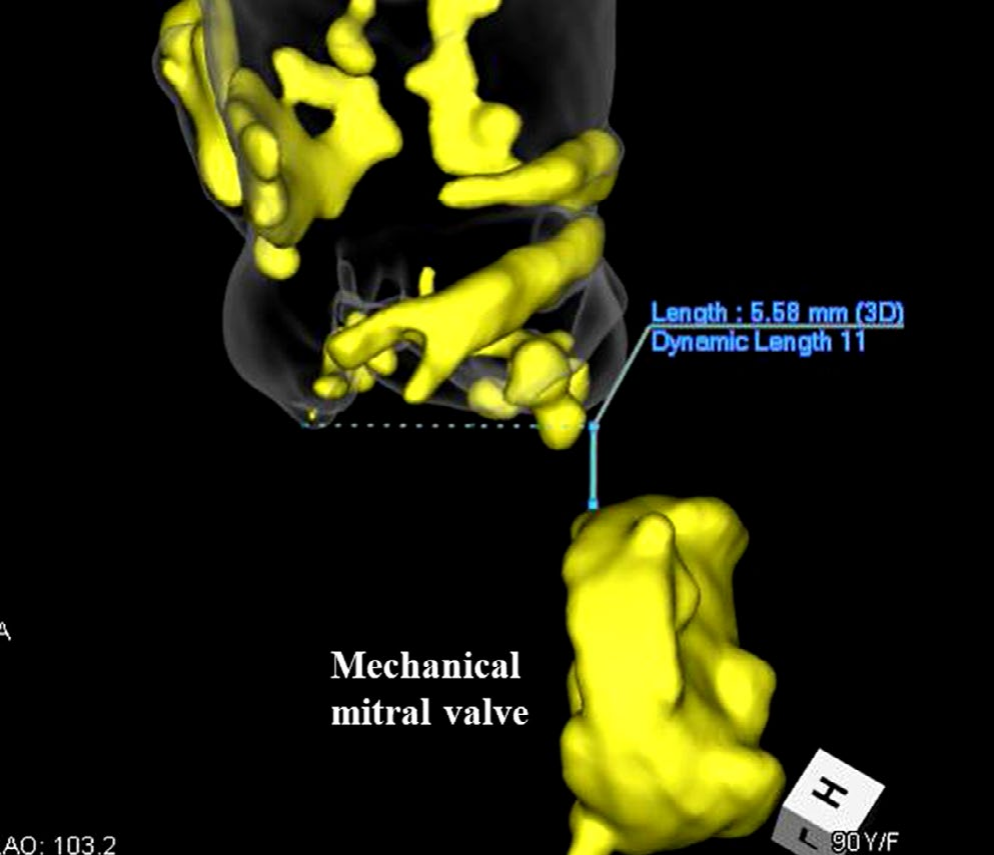
Preoperative three‐dimensional computed tomography images. Preoperative three‐dimensional computed tomography images reconstructed and converted from contrast‐enhanced multilayered image showed the distance between the aortic valve and the mechanical mitral valve (5.6 mm)

A 91‐year‐old woman was diagnosed with severe aortic stenosis and was admitted to our institution. She had a history of coronary artery bypass grafting (left internal thoracic artery to left anterior descending artery and mitral valve replacement using a mechanical valve; St. Jude Medical) at 71 years of age where the chordae tendineae connecting to the anterior leaflet were preserved, as well as percutaneous coronary interventions at 71 and 89 years of age. Transthoracic echocardiography indicated paradoxical low‐flow, low‐gradient aortic stenosis (mean gradient, 21 mm Hg; area, 0.7 cm^2^; annulus diameter, 18 mm), with a left ventricular ejection fraction of 63%. Preoperative contrast‐enhanced multilayered computed tomography (CT) scans showed an annulus area of 301 cm^2^ and diameter of 20 mm. Three‐dimensional CT images, reconstructed and converted from contrast‐enhanced multilayered images, revealed the distance between the virtual basal ring and the mechanical mitral valve was 5.6 mm (Figure 1). Contrast‐enhanced CT showed moderate stenosis and severe calcification of both iliofemoral arteries, which precluded transfemoral TAVI. The predicted operative mortality rates derived from the Society of Thoracic Surgeons Score and the logistic EuroSCORE were 9.5% and 48.2%, respectively.

**Figure 2:**
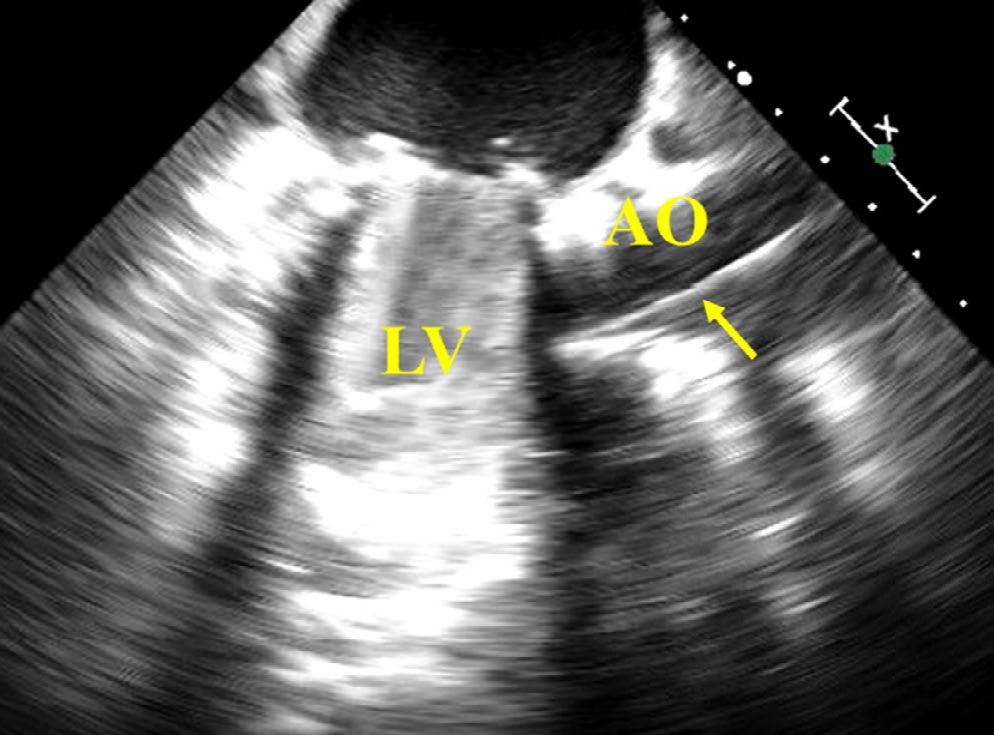
Intraoperative transesophageal echocardiography. Intraoperative transesophageal echocardiography showed the guidewire (arrow) located well away from the mitral prosthesis. AO, aorta; LV, left ventricle

**Figure 3:**
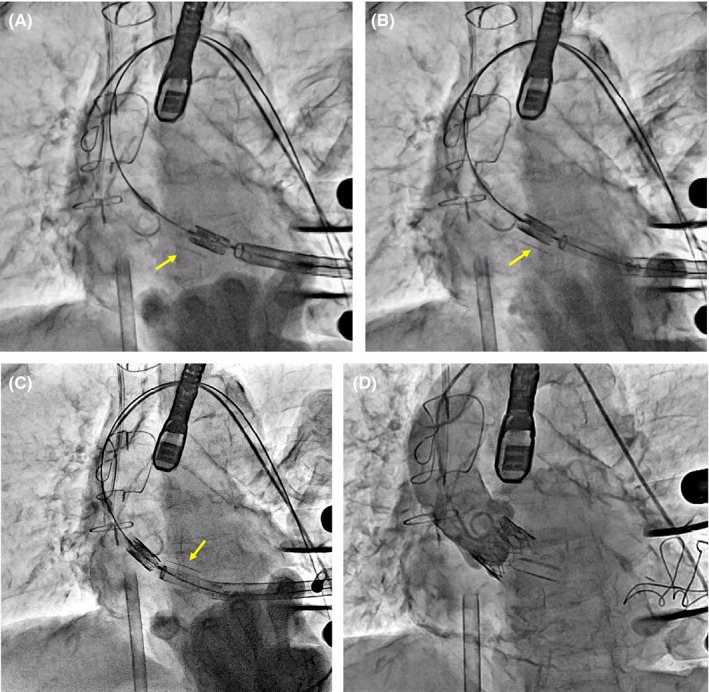
Operative findings. A, During delivery, the transcatheter heart valve became stuck in the left ventricle. B, The mitral prosthesis (arrow) moved parallel to delivery of the transcatheter heart valve. C, The spatial relationship between the transcatheter heart valve and the mitral prosthesis (arrow) changed after the aortic positioning. D, Postdeployment angiography showed trivial paravalvular leakage and no interference with the mitral prosthesis

Transapical TAVI was performed under general anesthesia using transesophageal echocardiographic and fluoroscopic guidance. After thoracotomy in the fifth intercostal space, two large purse‐string sutures with pledgets were placed at the left ventricular apex, and epicardial ventricular pacing wires were positioned near the sutures. Aortic root angiography was performed through a pigtail catheter in the left femoral artery. After puncturing the apex, a 260 cm, 0.035 in Amplatz Stiff Guidewire (COOK Medical) was positioned in the descending aorta, and a 24F Ascendra plus sheath (Edwards Lifesciences Inc) was inserted over the wire into the left ventricular apex. Balloon aortic valvuloplasty was not performed because of mild calcification and the wide area of the aortic valve. A 23 mm Edwards SAPIEN XT valve (Edwards Lifesciences Inc) was inserted through the sheath to the aortic annulus. Intraoperative transesophageal echocardiography showed the guide wire located well away from the mitral prosthesis (Figure 2). However, the THV became trapped in the left ventricle (Figure 3A and 3B). With Lunderquist Extra Stiff guidewire (COOK Medical) being inserted and repositioned as an alternative to the previous guide wire, the situation remained unchanged. Bleeding at the insertion site increased with changing the direction of the sheath to release the THV. Due to unstable hemodynamics, extracorporeal membrane oxygenation support was initiated using the right femoral artery and vein. Once the patient was hemodynamically stable, the THV was forced into the aortic valve and released (Figure 3C). The THV was then positioned in the aortic annulus and unfolded under rapid pacing. Postdeployment angiography showed the THV was positioned a little lower than the intended position with trivial paravalvular leakage and normal leaflet motion of the mitral prosthesis (Figure 3D). The patient was discharged on the 27th postoperative day without symptoms.

## 3 DISCUSSION

A major concern for TAVI in patients with mitral prosthesis is the potential for mechanical interference between the THV and the mitral prosthesis. Although the aortic annulus to the mitral prosthesis seems to be a risk factor for interference, a recent meta‐analysis of case reports and case series of TAVI in this complex setting proved that it was not related to procedural success.^5^

We reported transapical TAVI after mitral valve replacement using mechanical valve. In this case, the THV became trapped in the left ventricle during delivery despite the relatively wide distance between the aortic annulus and the mitral prosthesis. Additionally, intraoperative transesophageal echocardiography showed the guidewire was positioned well away from the mitral prosthesis. The trick of this case is that the THV was caught in the anterior chordae tendineae preserved anteriorly in the previous operation, which is difficult to discriminate by CT and/or echocardiography. This is why the mechanical mitral valve moved parallel to the THV and the positional relationship changed before and after the aortic positioning without its dysfunction. We dealt with this problem by forcing the THV into the aortic valve for the release. From the retrospective view of this case, inserting a sheath just below the aortic valve for delivery of the THV might be proposed as an alternative to avoid the chordae tendineae.

In conclusion, transapical TAVI after mitral valve replacement was challenging because the THV became trapped in the anterior chordae tendineae of the left ventricle. As the problem was resolved by forcing the THV into the aortic valve in this case, inserting a sheath beneath the aortic valve would be proposed as an alternative.

## CONFLICT OF INTEREST

The authors declare no conflicts of interest.

## AUTHOR CONTRIBUTIONS

Akihisa Furuta: study concept/design, drafting article and critical revision, approval of article. Katsumasa Sato: study concept/design, critical revision, and approval of article. Hironobu Morimoto: study concept/design and critical revision. Shogo Mukai: critical revision and approval of article. Kenji Goto: data interpretation and approval of article.
